# The immune system and metabolic products in epilepsy and glioma-associated epilepsy: emerging therapeutic directions

**DOI:** 10.1172/jci.insight.174753

**Published:** 2024-01-09

**Authors:** Shashwat Tripathi, Cody L. Nathan, Matthew C. Tate, Craig M. Horbinski, Jessica W. Templer, Joshua M. Rosenow, Timothy L. Sita, Charles D. James, Benjamin Deneen, Stephen D. Miller, Amy B. Heimberger

**Affiliations:** 1 Department of Neurological Surgery,; 2 Malnati Brain Tumor Institute of the Robert H. Lurie Comprehensive Cancer Center,; 3 Department of Neurology,; 4 Department of Pathology, and; 5 Department of Radiation Oncology, Feinberg School of Medicine, Northwestern University, Chicago, Illinois, USA.; 6 Department of Neurosurgery, Baylor College of Medicine, Houston, Texas, USA.; 7 Department of Microbiology-Immunology, Feinberg School of Medicine, Northwestern University, Chicago, Illinois, USA.

## Abstract

Epilepsy has a profound impact on quality of life. Despite the development of new antiseizure medications (ASMs), approximately one-third of affected patients have drug-refractory epilepsy and are nonresponsive to medical treatment. Nearly all currently approved ASMs target neuronal activity through ion channel modulation. Recent human and animal model studies have implicated new immunotherapeutic and metabolomic approaches that may benefit patients with epilepsy. In this Review, we detail the proinflammatory immune landscape of epilepsy and contrast this with the immunosuppressive microenvironment in patients with glioma-related epilepsy. In the tumor setting, excessive neuronal activity facilitates immunosuppression, thereby contributing to subsequent glioma progression. Metabolic modulation of the IDH1-mutant pathway provides a dual pathway for reversing immune suppression and dampening seizure activity. Elucidating the relationship between neurons and immunoreactivity is an area for the prioritization and development of the next era of ASMs.

## Introduction

Epilepsy affects approximately 1% of the population and can lead to a substantial decrease in quality of life and death in up to 5% of patients (1). Current treatment options focus on symptomatic control of neuronal hyperactivity. Despite the development of numerous antiseizure medications (ASMs), nearly one-third of patients with epilepsy have drug-resistant epilepsy (2). The preponderance of epilepsy research has focused on altering neuronal activity, but there have been several exceptions focused on immunomodulation (3–5). Clinical evidence for the role of neuroinflammation in epilepsy includes the use of corticosteroids in super-refractory epilepsy and the antiinflammatory effects of several common ASMs (6–8). Longitudinal profiling of preclinical murine models such as pilocarpine-induced status epilepticus (SE) similarly implicate the immune system in epilepsy by demonstrating a transient increase in activated CD11b^+^ and F4/80^+^ macrophages followed by an increase in CD3^+^ T cells in the brain (9). Genetic and pharmacological manipulation of immunoreactivity also influences seizure prevention, threshold, frequency, and induction in preclinical models (10–13). More specifically, IL-1 receptor (IL-1R) upregulation has been noted after seizure activity, and Toll-like receptor (TLR) activation results in changes in synaptic transmission and long-term potentiation (LTP) (11). Reduced Ca^2+^ current repolarization and γ-aminobutyric acid (GABA) activity is associated with IL-1B activation (14). Furthermore, astrocytes have been shown to increase the excitatory neurotransmitter glutamate following IL-1R/TLR pathway activation (15). Within the emerging field of metabolomics, many of which have immunomodulatory properties, there is increasing evidence that the immune system may be modulating epilepsy.

## Types of human epilepsy

**Figure 1:**
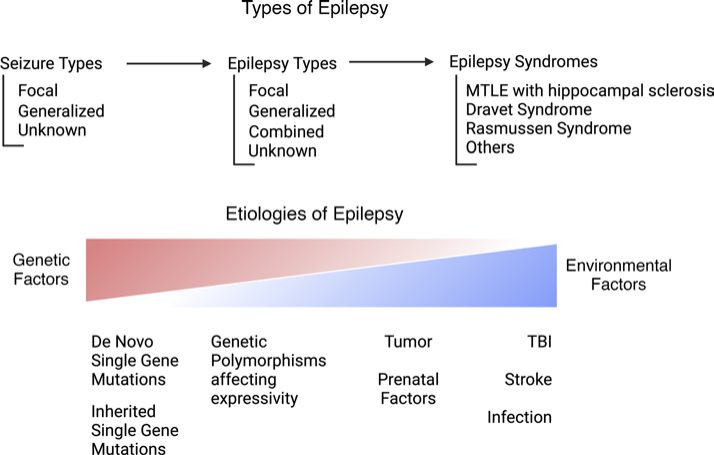
Schematic overview of epilepsy diagnosis and common etiologies of epilepsy, from genetic to environmental. TBI, traumatic brain injury.

Human epilepsy encompasses a spectrum of heterogeneous etiologies, with causality spanning from genetic to environmentally induced disease (Figure 1). The International League Against Epilepsy (ILAE) classifies epilepsy based on three diagnostic levels, including seizure type, epilepsy type, and epilepsy syndrome (16). Seizure type is based on what part of the brain is involved. Focal seizures originate in one hemisphere, whereas generalized seizures involve bilateral hemispheres and can be of unknown origin, with insufficient data to discern onset. For epilepsy type, it is critical to determine whether an initial seizure is provoked or unprovoked. A provoked acute symptomatic seizure may be attributed to an identifiable cause, such as a drug, toxin, metabolic disturbance, or acute structural injury. An unprovoked seizure occurs in the absence of an inciting factor or more than one week after an acute insult/injury. Epilepsy syndrome is the third level of identification and is based on a constellation of signs, symptoms, and diagnostic tests that are common to a specific disorder. Key diagnostic information includes clinical history, seizure type(s), electroencephalography, and imaging. These syndromes are more commonly identified in childhood and have a broad range of etiologies. The identification of an epilepsy type and syndrome can help inform individuals as to their risk of potential associated comorbidities such as intellectual disability, psychiatric symptoms, sleep disturbances, and more.

The underlying etiology of a person’s epilepsy, such as structural, genetic, infectious, metabolic, immune, unknown, or a combination thereof, guide treatment and prognostication (16). Despite advances in diagnostic testing, up to one-third of patients with epilepsy have an unknown underlying etiology (16). The most common surgically treated epilepsy syndrome is mesial temporal lobe epilepsy (MTLE), often associated with hippocampal sclerosis (16). Although this syndrome may be an acquired cause of epilepsy, certain genetic factors may contribute to the development of hippocampal sclerosis (17, 18). Other acquired structural pathologies that can result in a seizure include stroke, traumatic brain injury, tumor, or CNS infections (e.g., herpes simplex virus). Some structural abnormalities may be genetic, such as malformations of cortical development. Genetic epilepsies are associated with known or presumed genetic mutations that may be complex and involve multiple genes and/or environmental factors. In certain circumstances, such as tuberous sclerosis or focal cortical dysplasia, seizures are due to a combination of genetics and structural lesions. Metabolic disorders due to genetic aberrations may also ultimately result in disruption of biochemical pathways. Downstream effects of abnormal metabolism include accumulation of toxic substances and disruption of electrolyte levels that may lead to seizures.

The immune system likely contributes to the development of epilepsy, with some types of epilepsy having a clear connection to immune dysfunction, such as paraneoplastic encephalitis and autoimmune encephalitis (e.g., NMDA receptor encephalitis). Over the last decade, studies have implicated the immune system in other types of epilepsy and syndromes that were not traditionally attributed to the immune system by the ILAE (19–21); however; in 2017, the ILAE introduced a new classification of “autoimmune epilepsy” in light of the growing research recognizing the importance of immune etiologies for epilepsy.

## Immunological reactivity in human epilepsy

There is a paucity of human data regarding immune cell profiling in patients with epilepsy and existing studies often include various epilepsy types. The prototypical example of inflammatory epilepsy is Rasmussen encephalitis, which is possibly triggered by autoimmunity or a yet to be identified virus (22, 23). Analysis using immunohistochemistry, flow cytometry, and T cell receptor sequencing of brain tissue from Rasmussen patients revealed an activated and proinflammatory T cell response (24). More recent studies spanning various types of epilepsy such as Sturge Weber, focal cortical dysplasia, temporal lobe epilepsy, etc., identified an activated microglial population that may be interacting with T cells based on physically interacting cell sequencing analysis (20). However, a limitation of this study was the use of postmortem brain, which likely had degradation of mRNA subgroups (25). Additionally, this study included a heterogeneous epilepsy population, with variable immunoreactivity skewing specific immune signatures. Other studies using human samples have found a substantial T cell population within epileptic pediatric patients. Flow cytometric analysis of brain specimens from a variety of pediatric epilepsy patients (*n* = 33, including MTLE, focal cortical dysplasia, encephalomalacia, and Rasmussen encephalitis) found frequent myeloid cell and CD4^+^ and CD8^+^ T cell infiltration (19). Deeper analysis of myeloid cell composition showed that activated blood-derived immune cells, but not microglia, correlated with seizure frequency. Most strikingly, γδ T cells were frequently identified and directly correlated with disease severity, whereas Tregs inversely correlated with disease severity (19). Preclinical mouse models corroborated these results. Mass cytometric approaches revealed increased IL-17–producing CD4^+^ and CD8^+^ T cells, with decreased LAG3^+^CD8^+^ T cells in the peripheral blood (21). Immunohistochemistry of nine patients with MTLE displayed increased activation of microglial cells and vessel-associated leukocytes (26). However, this analysis was confounded by the lack of a microglia-specific marker and relied on morphology and CD45^+^ staining, which cannot discriminate between peripherally derived immune cells. An immunohistochemistry-based study of cortical tissue from 13 patients with various epileptic syndromes found increased IL-1B, IL-8, IL-12p70, and MIP-1b in the epileptogenic focus (27). It is likely that there are significant differences in the types and extent of immunoreactivity between the various etiologies of epilepsy and clarification of immunomodulatory targets would provide supplementary strategies for therapeutic manipulation. There is insufficient information at this time to guide clinicians to specific precision medicine immunomodulatory strategies, because the immune characterization and profiling of targets have not yet been done in a sufficiently rigorous fashion to make a recommendation.

## Preclinical murine models of epilepsy

Multiple preclinical murine models of epilepsy have been developed to study the pathophysiology of both acute and chronic epilepsy and to evaluate potential novel treatment approaches. Kainic acid (KA) and pilocarpine are the two most common chemoconvulsants used to study chronic epilepsy (28). The KA and pilocarpine models were designed to mimic temporal lobe epilepsy (TLE) and display a similar clinical course and pathological findings as patients with chronic epilepsy (28–31). Specifically, these models show that an initial injury of the hippocampus/temporal lobe causes SE, with a latent period before spontaneous seizures that are followed by chronic spontaneous recurrent seizures. Histology in the preclinical models closely recapitulates the disease within human patients. The KA model causes recurrent seizures, with hippocampal sclerosis. The damage in the KA models is restricted to the hippocampus, which can be beneficial for studying behavioral changes; however, when seizures spread in patients with MTLE, the entire temporal lobe may be involved. There is age-dependent seizure penetrance in these models, with younger animals being more susceptible to KA, which must be considered when modeling pediatric versus adult MTLE. The pilocarpine model resembles human hippocampal seizures and shows similarities in affected networks and the neurochemical changes observed in patients with TLE (32–34). Importantly, pilocarpine administration causes neocortical lesions. While pilocarpine induces a more uniform spontaneous seizure frequency compared with KA, the duration of the SE is still variable, ranging between 30 minutes and 2 hours, which may affect immune cell infiltration following the initial induction. Shared limitations for both models include time constraints to reach chronic epileptic states, high mortality, and heterogeneity in spontaneous seizure frequency and severity. In both models, the seizure-inciting etiology is known but in humans, the initial injury is often unknown and the immune infiltration may be a component of the initial inducing event, which is not currently modeled in animals. Similarly, the temporal dynamics cannot be fully captured by such models given the heterogeneity and uncertainty in humans. In addition to induced chronic epilepsy models, acute seizure models are typically used for drug screening, where ASMs are commonly given one hour before electroshock and seizure frequency is measured (35). These models may be appropriate for testing seizure-terminating drugs but their utility in assessing immune therapeutics is uncertain, as these drugs would likely be intended to modulate the development of chronic epilepsy.

## T cell responses during acute epilepsy

T cell brain invasion has been noted in mice with KA-induced epilepsy and tissue obtained from patients with TLE (26). However, a recent single-cell analysis of RNA from human epileptic tissue showed few T cells, corroborating prior immunohistochemical analysis of the hippocampus from patients with TLE (20, 36). In RAG1-KO mice, which lack T and B cells, KA administration increased neurodegeneration and resulted in earlier onset of seizures, suggesting a potential neuroprotective effect of T cells (26). In KA murine models, γδ T cell– and IL-17RA–deficient mice had decreased seizure activity compared with Treg-depleted mice, with increased seizure activity, thus highlighting immune modulation as a potential therapeutic option (19). Immune profiling of children with febrile infection–related epilepsy syndrome (FIRES) found decreased circulating naive T cells and Tregs and impaired TLR responses in peripheral blood (37). However, the role of T cells might not be so clear, as a recent study using OT-1/*Rag1*^–/–^ mice found that CD8^+^ T cells exert neurotoxic effects on hippocampal neurons, which lead to TLE secondary to limbic encephalitis (38).

Differences noted between human and murine models may be attributed to the time point analyzed. Human tissue obtained during routine epilepsy surgery is acquired from patients who have failed multiple ASMs and are in the chronic stage of the disease. In contrast, murine model analysis is typically done in a more acute setting. Even in models such as pilocarpine and KA that attempt to recapitulate the chronic setting, mice are analyzed after just a couple weeks of seizures relative to the many years of seizures patients often endure before undergoing epilepsy surgery.

## Microglia surveil and prune neuronal activity

**Figure 2:**
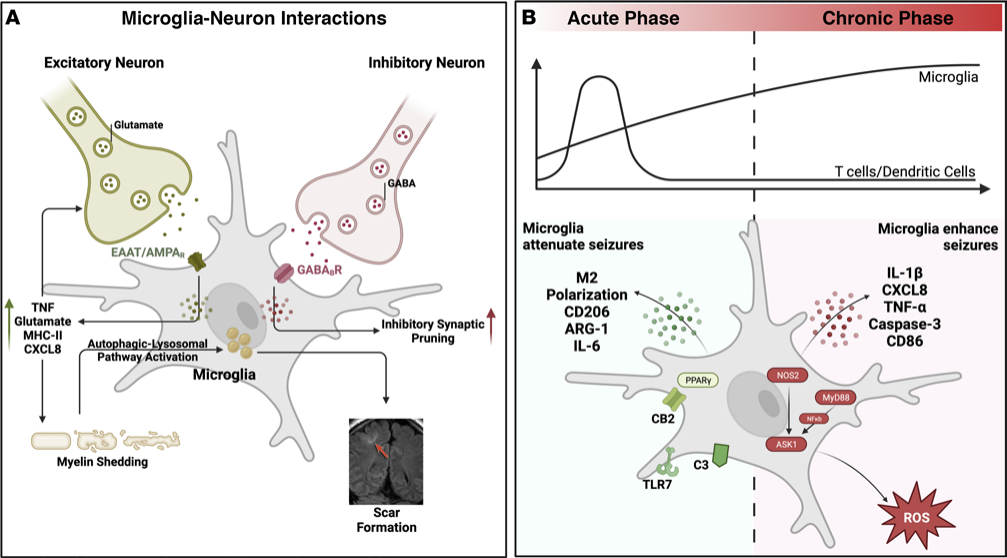
Proinflammatory microglia facilitate chronic epilepsy. (**A**) Microglia facilitate chronic epilepsy through direct and indirect interactions with excitatory and inhibitory neurons, leading to sustained aberrant electrical activity and scar formation. Microglia interact with both excitatory and inhibitory neurons. During development, signaling through GABA_B_ receptors leads to inhibitory synaptic pruning. Microglia also have various glutamate receptors on their surface, including EAAT and AMPA. Signaling through these receptors leads to production of proinflammatory cytokines, which promote increased glutamate release from presynaptic excitatory neurons and damage to the cortex. Toxic damage to surrounding axons causes myelin shedding, which through autophagic pathways ends up in lipid droplets within the microglia and leads to scar formation and lesional epilepsy. (**B**) The temporally distinct dual role of microglia. Acutely, M2-like microglia attenuate seizure activity through the production of immunosuppressive cytokines along with upregulation of cell surface receptors. Chronically, epileptic microglia may become proinflammatory, releasing cytokines into the microenvironment that facilitate chronic epileptic states.

Microglial cells are the primary resident immune cells of the brain surveying the CNS to remove debris and direct proinflammatory responses. During CNS development, synapses are initially overproduced and then pruned based on neuronal activity. Microglia are crucial for healthy neuronal development and synaptic maturation, where they play a major role in this synaptic pruning (39–41). While the mechanism of synaptic pruning has not been fully elucidated, the CX3CR1/CX3CL1 pathway has been implicated, as neurons upregulate CX3CL1 during maturation. Indeed, CX3CR1-KO mice have higher numbers of synapses and a higher frequency of miniature excitatory postsynaptic currents. Even after development, microglia can sense glutamate and GABA concentrations in the extracellular matrix through various glutamate transporters (EAAT/SLC1A3 and xCT) and GABA receptors (GABA_B_Rs) (42–44). Of note, expression of these transporters relies heavily on the location and activation state of the microglia. Microglia upregulate these transporters, and chronic activation of these receptors and binding of glutamate onto microglial AMPA and kainate receptors leads to the release of proinflammatory cytokines like TNF (Figure 2A). A feedforward loop can be set up through glutamate activity in which microglia may also secrete additional glutamate and TNF (42, 45–47). Microglia express metabotropic glutamate receptors, including mGluR2/3/5. Activation of mGluR2 promotes neuroinflammation through the release of inflammatory cytokines and glutamate; in contrast, mGluR5 activation is neuroprotective (28–50). However, the expression of mGluRs on microglia may be limited, and the physiological role of these receptors still needs to be elucidated. Additionally, activation of microglia leads to increased MHC-II expression and glutamate release, potentially leading to a toxic feedback loop that increases neural activity, microglial activation, and aberrant synaptic pruning (42–50).

Microglia are capable of sensing GABA through GABA_B_R and a unique population of GABBR1- and GABBR2-expressing microglia specifically prune inhibitory (GABAergic) synapses, thus providing a potential mechanism for microglia-dependent epileptic activity through reduction of miniature inhibitory postsynaptic currents. These studies focused on understanding the role of microglia in normal brain development but also suggest that deficits in microglia might contribute to neurological disorders that involve aberrant electrical and synaptic activity, i.e., epilepsy. For example, chronic immune activation and neural signals can promote excessive pruning, leading to destruction in synaptic architecture and suggesting that reducing microglial synaptic pruning has therapeutic potential (51). Interestingly, conditional KO of GABBR1 on CX3CR1 cells did not result in increased KA sensitivity in the acute setting; however, the chronic epileptic phase of KA administration was not tested in these mice (43, 44).

## Brain-resident microglia likely support chronic epilepsy

Given the ability of microglia to preferentially prune either excitatory or inhibitory receptors based on expression and activation states, aberrant microglial activity may promote epileptogenic activity. The role of microglia as either pro- or antiepileptic is controversial and may be a function of distinct phenotypic morphology and activation states based on distance from epileptic foci and chronicity/type (52–54). In the epileptogenic foci and sclerotic areas, activated proepileptic microglia are the most frequent (26). Morphological, functional, and transcriptomic analyses show increased activated microglia with companying proinflammatory cytokines in sclerotic areas of the hippocampus compared with nonsclerotic areas (53). Murine models are consistent in showing increased IL-1β, CXCL8, and TNF-α after seizures in the focal areas. Microglia have been shown to produce elevated ROS levels in both SE and other refractory epilepsy models (54–58). In pilocarpine-induced epilepsy models, NOX2 is the main source of ROS and is produced by microglia (59). ROS have been shown to support LTP and long-term depression of neuronal activity through activation of various protein kinases, including those of the ERK pathway (60, 61). MyD88, which promotes cytokine secretion and subsequent peripheral immune cell recruitment, is another key pathway implicated in the epileptogenic role of microglia (62, 63). Inhibition of MyD88 increased microglia polarization to the M2 phenotype and improved outcomes after SE (64). Several other proinflammatory modulators on microglia may dampen seizure activity. Apoptosis signal–regulating kinase 1 (ASK1) participates in inflammation, apoptosis, and cell proliferation (65). Studies have shown increased levels of ASK1 in hippocampal slices from patients with epilepsy and in preclinical rat models (66, 67). ASK1 is highly expressed in microglia and macrophages during the acute phase of epilepsy, and ASK1 KO reduced seizure activity in the acute phase and the frequency of spontaneous recurrent seizures in the chronic phase (65). Cumulatively, these data indicate that microglia might have a role in prolonging neuronal hyperexcitation and chronic epileptic states.

While most literature suggests a proepileptogenic role for microglia, microglia may also suppress abnormal neuronal activity in SE, particularly in the acute phase (Figure 2B) (54, 68, 69). Neurogenesis has been noted in preclinical MTLE models, and the role of microglia in synaptic pruning and engulfment of new granule cells might be beneficial in curbing the creation of aberrant neuronal connections that lead to abnormal electrical activity and seizures (69, 70). Cannabinoid compounds, such as *N*-acylethanolamine palmitoylethanolamide (PEA), have also been investigated as potential antiepileptic therapeutics (71–77). The mechanism of PEA is still unclear; however, leading hypotheses include potential immune system–related effects such as downregulation of mast cell activation and direct and indirect cannabinoid receptor (CB1 and CB2) activation (78). Notably, CB2 is highly expressed in various immune cells (79, 80), including reactive microglia where its activation may suppress reactivity and have a neuroprotective effect. Additionally, TLR9 activation on microglia in KA seizure models attenuates neurogenesis (81). Similarly, rosiglitazone, a PPARγ agonist, increased M2 polarization and prevented neuronal loss in pilocarpine-induced TLE mouse models (82). Further evaluation of the activation state of microglia might provide insight into their potentially conflicting roles in epilepsy, with evidence than an M1 phenotype decreases neurogenesis, while an M2 phenotype promotes neurogenesis (83–85). Therefore, activation and polarization of M1-like microglia early in epilepsy warrants further investigation. This discrepancy in microglial function may be due to differences in kinetics, with a more proinflammatory role during the active or early stages of epilepsy and an immunologically suppressive role during later chronic epilepsy. A key confounder in the analysis of innate immunity is the marked heterogeneity in cell surface markers used to classify macrophages, monocytes, and microglia that may also lead to contradictory results.

## Microglia as a function of epilepsy type

Neurotoxicity and chronic inflammation lead to demyelination and cell death. Microglia, and to a lesser extent peripheral immune cells, are the main cells responsible for debris removal (86). Through the autophagic lysosomal pathway, microglia degrade engulfed debris containing lipid-dense myelin fragments; however, accumulation of these lipid droplets impairs microglial function and is toxic (86–88). In multiple sclerosis models, microglia likely contribute to lesion formation. The inhibition of the autophagic lysosomal pathway in microglia or supplementation with conjugated linoleic acid attenuates inflammation and may improve recovery from CNS demyelination injuries, thereby improving neurologic outcomes (89, 90). Given the abundance of activated immune cells specifically localized to the seizure focus in epileptic tissue, toxic autophagic lysosomal clearance of myelin debris may occur, possibly leading to structural lesions associated with epilepsy. The prevalence of immune cells in lesions also suggests a potential difference in the immune activation states between lesional and nonlesional epilepsy and awaits future analysis.

## Context-dependent role of peripherally derived monocytes in seizures

Peripherally derived immune cells such as monocytes and macrophages also contribute to the proinflammatory signature seen in patients with epilepsy (21, 91–93). While monocyte infiltration in the brain is associated with viral encephalitis–induced seizures, monocyte inhibition did not prevent hippocampal damage. Therefore, monocytes might contribute to seizure activity but not the cortical damage seen in chronic epilepsy (94). Further studies showed that the depletion of CCR2^+^ monocytes following SE was neuroprotective and reduced blood-brain barrier (BBB) disruption (27, 95–98). However, a recent report found that CCR2 ablation did not affect seizure susceptibility and inflammation in preclinical pediatric traumatic brain injury models, highlighting a potential disease-specific role of CCR2 and seizures (99). The role of peripheral immune cells is also likely model dependent. In pilocarpine models, SE onset requires induction of peripheral inflammation, and antagonism of peripheral inflammation interferes with this phenotype (100, 101). In contrast, KA, an analog of glutamate, works directly on neurons to induce SE, with immune inflammation occurring after SE onset (102).

## Transient CD11C^+^OX62^+^ DC infiltration after seizure

While most prior work has focused on the role of microglia and astrocytes in epilepsy, given their direct role in synaptic homeostasis in healthy parenchyma, several reports have suggested a potential role for CD11c^+^ cells in proinflammatory immune responses following SE. These cells may be DCs, but additional marker validation is needed for verification. At steady state, there is a lack of detectable DCs in the brain parenchyma due to the BBB (103). However, DCs have been reported within the CNS in many infections and autoimmune conditions when the BBB has been disrupted, thus providing rationale for a potential role of DCs in epilepsy (104–106). In pilocarpine-induced seizure models, immunohistochemistry demonstrated CD11c^+^ cells concentrated in the hippocampus, thalamus, and temporal cortex one day after SE (107). In these models, OX62^+^ DCs peaked three days after SE and by day 12, the parenchyma was devoid of OX62^+^ DCs, similar to baseline. This pattern of OX62^+^ DCs mirrors the temporal increase in CD11c^+^ DCs in the same model and mirror the temporal pattern of lymphocytes (107–109). To look for evidence of direct DC–T cell interactions, the investigators tried to find colocalization of OX62 with T cell surface markers, but none were found (109). However, DC–T cell interactions could still be playing a role in epilepsy through soluble factors, such as inflammatory cytokines, or these interactions may be present but not within the epileptic foci. Total body–irradiated mice were examined to better understand the origin of the DCs in the brain after KA administration. Mice undergoing total body radiation before induction of an excitotoxic lesion showed decreased infiltration of OX62^+^ DCs in the brain, indicating that these DCs are peripheral blood derived (109). The unique timeline of DC recruitment raises questions regarding their function in the immune response following SE (Figure 2B). As DCs appear more than 24 hours after initial seizure events, they are unlikely to contribute to the acute response after SE but may facilitate chronic epileptic states in these models. Unfortunately, these studies only examined the cortex following one seizure and did not report on the chronic epileptic state where mice experience several spontaneous recurrent seizures per day.

## Glioma-related epilepsy

### Interplay between neurons, glioma cells, and microglia.

**Figure 3:**
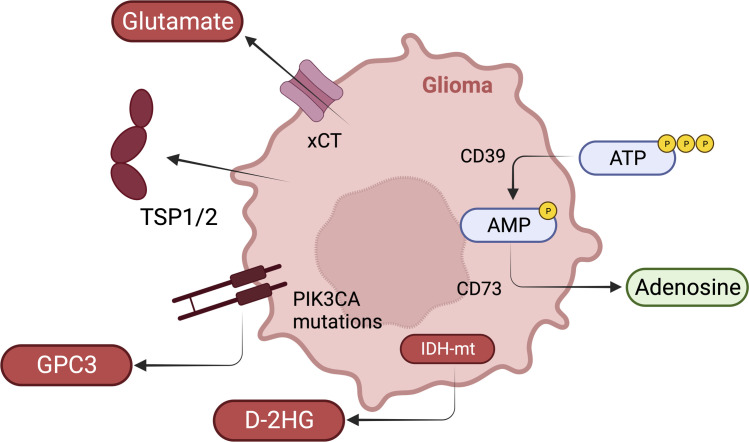
Major glioma-intrinsic mechanisms of epileptogenesis. The TME of GRE is distinct from other forms of epilepsy due to the presence of glioma cells. The metabolic landscape includes high adenosine concentrations achieved through the CD39/CD73 pathway expressed within the TME, which is immunosuppressive but also a seizure suppressant. On the other hand, several proepileptic pathways exist on glioma cells, including (i) aberrant glutamate flux through xCT channels; (ii) secretion of TSP1/2, leading to excitatory stimulation; (iii) secretion of GPC3 from PIK3CA-mutant gliomas, which inhibits normal KCC2 channels on peritumoral neurons; and (iv) the presence of the oncometabolite D-2HG in IDH-mutant (IDH-mt) gliomas, which directly increases neuronal activity.

Brain tumor patients often clinically present with epilepsy, and depending on tumor type, over 40% of patients have glioma-related epilepsy (GRE) (110). Distinct from the general proinflammatory microenvironment in non-GRE, the glioma tumor microenvironment (TME) is for the most part immunosuppressive (111). An understanding of this apparent discrepancy between similar electrical activity providing/assisting to create two opposing environments may provide important insights into both diseases. There are likely other differences in the underlying microenvironments, such as metabolites and cellular interactions, that ultimately arbitrate aberrant neuronal firing (Figure 3).

## Neuron-glioma synapse

Direct and indirect functional synapses between neurons and glioma cells have been found in high-grade gliomas (111–115). Neuronal release of activity-dependent soluble factors, such as glutamate, neuroligin-3 (NLGN3), and brain-derived neurotrophic factor (BDNF), promote glioma progression (115–117). Within the neuron-glioma synapse (NGS), glioma cells express AMPAR that binds to glutamate, leading to increased proliferation and invasion. Monocyte-produced metalloprotease (MMP) ADAM10 causes the release of soluble synaptic protein NLGN3, which, through the PI3K/mTOR pathway, induces glioma growth. Reciprocally, glioma progression can mediate neuronal hyperexcitation through mass effect and molecular mechanisms. Glioma cells secrete aberrant glutamate through the xCT glutamate/cystine antiporter, leading to glioma progression and hyperexcitation on surrounding peritumoral neurons (118). Additionally, secretion of TSP2 promotes peritumoral excitatory synapse formation through the a2δ-1/Rac1 signaling pathway (119, 120). Glioma-derived MMPs break down the perineuronal nets (PNNs) that stabilize inhibitory interneurons (121). Glioma-specific mutations in PIK3CA have been implicated in seizure onset, with glypican-3 (GPC3) emerging as a driver of synaptogenesis and hyperexcitation. GPC3 from gliomas impairs KCC2 channels in neurons, leading to a paradoxical depolarization response to GABA. KCC2 impairments have been linked to Src/TrkB-mediated pathways that are secondary to increased neuronal Zn^2+^ concentrations. Reduced KCC2 may also be related to prolonged NMDAR activation from the increased glutamate pool (122). Finally, BDNF, secreted by neurons and microglia, decreases the surface expression of KCC2 through Src/TrkB pathways (123, 124).

## Neuron-microglia interaction

Microglia infiltration correlates with late spontaneous seizure activity in murine models. As discussed above, the role of microglia in synaptic pruning and formation in healthy and disease states supports potential mechanisms by which microglia might alter the NGS. Microglia may mediate NGS through direct neuronal connections as seen in epilepsy models and/or through the secretion of soluble proteins such as MMPs (PNN breakdown), BDNF, IL-10, and TGF-β, which have all been shown to increase hyperexcitation (117). Activation of TGFβR1 increases glutamate and K^+^ concentrations through surface expression of aquaporin 4 (AQP4), thus promoting hyperexcitability and immunosuppression on the microglia themselves. It has also been hypothesized that microglia express the xCT antiporter, which might contribute to the large glutamate pool in the peritumoral environment (125). Reciprocally, spreading depolarizations in peritumoral neurons have been linked to microglia recruitment (111).

## Isocitrate dehydrogenase–mutant glioma-associated seizures: convergence of metabolomic and immune modulation

Patients with gliomas with isocitrate dehydrogenase (IDH) mutations are more likely to present with seizures, because the metabolic product of mutant IDH, D-2-hydroxyglutarate (D-2HG), synchronizes neuronal activity in a similar manner to the hypersynchronous neuronal firing that drives seizures (126). D-2HG levels are low in cells and tissues and becomes elevated 10- to 100-fold in IDH-mutant gliomas. D-2HG acts as a competitive inhibitor of many enzymes, including dioxygenases that demethylate DNA and histones (127, 128). Thus, the D-2HG–mediated inhibition of such demethylases gradually shifts the IDH-mutant cell toward DNA and histone hypermethylation. This hypermethylated state in immune cells prevents differentiation and effector T cell functions (129, 130). The addition of D-2HG to murine primary neuron-glia cultures produces a distinct seizure pattern compared with that generated when only glutamate is added to the same culture, suggesting that while D-2HG is a glutamate analog, its epileptogenicity is not simply through activation of conventional glutamate receptors (126). As such, the underlying mechanism of D-2HG in seizure production has not yet been elucidated. D-2HG also has an immunological role, as it is taken up by CD8^+^ T cells and reduces their cytotoxicity and impairs IFN-γ signaling by inhibiting glycolysis. D-2HG accumulates in the latter stages of macrophage activation and ultimately serves to inhibit inflammatory macrophage responses (129, 131). Myeloid cells become tolerized in the IDH-mutant TME through the re-orchestration of tryptophan metabolism, resulting in activation of the aryl hydrocarbon receptor. Ultimately, the pharmacological inhibition of tryptophan metabolism can partially reverse immunosuppression (132). The cytokine modulatory properties of D-2HG also inhibit microglial activation via the AMPK/mTOR/NF-κB pathway and IL-12 secretion from DCs (133, 134). Other immunoregulatory functions include inhibiting the activation of complement by both the classical and alternative pathways, attenuating complement-mediated tumor cell damage, decreasing cellular opsonization, and impairing complement-mediated phagocytosis (135). As such, therapies that target IDH1 mutations and downstream metabolic products, beyond having direct effects on delaying progression-free survival in low-grade gliomas (136), need to be considered in the context of immunomodulatory strategies. As IDH-mutant inhibitors decrease seizure frequency in mice bearing IDH-mutant gliomas (126), future clinical trials with IDH-mutant inhibitors (137) should include seizure frequency outcomes as a priority endpoint.

## The role of adenosine metabolism in GRE

Adenosine is an endogenous antiseizure metabolite that can acutely terminate seizure activity by directly inhibiting presynaptic action potentials (138, 139). Adenosine clearance occurs through the activity of adenosine kinase (ADK) to form AMP (140, 141). Astrocytes upregulate ADK in response to chronic activation and seizure activity (142, 143), which is observed in patients with MTLE (144, 145). Paradoxically, in animal models, overexpression of ADK alone can lead to seizure activity (146, 147). While the epileptic microenvironment is adenosine depleted through upregulation of ADK due to astrogliosis, the glioma TME is adenosine rich, which would therefore dampen epileptic activity. High adenosine concentration within the glioma TME occurs through hydrolyzation of extracellular ATP to AMP by CD39 and then into adenosine by CD73-expressing cells. In this context, adenosine has been shown to promote gliomagenesis through increased immunosuppression by binding to A2 adenosine receptors expressed on immune cells within the glioma TME (148, 149). This adenosine production triggers the generation of immunosuppressive IL-10, arginase 1, and TGF-β, while decreasing proinflammatory IFN-γ and TNF-α. These data would suggest that an adenosine-poor environment in the epileptic brain would not be conducive for the development and growth of gliomas.

## Development of immunomodulatory strategies for epilepsy

Cumulatively, these data provide rationale for considering modulation of the immune system and cytokines beyond the standard clinical practice of steroids in the treatment of some epilepsies. Prioritization of preclinical immunotherapeutic and cytokine targets should include (a) high expression and frequency of the gene/cell target that will be modulated, (b) a well-defined causal role in epileptogenesis, (c) selection of appropriate preclinical model(s) that recapitulate the human biology, and (d) a clinically feasible route of administration. The difference in T cell infiltration in mouse models versus humans is likely secondary to the kinetics of the immune responses. Although the brains of mice can be sampled during acute phases of epilepsy initiation, human brain specimens are usually only obtained during chronic epilepsy. The evidence seems to indicate that T cell–directed therapies may have a larger impact if administered early in the disease course and could potentially alter the chronicity of the disease. Notably, ASMs are largely effective and safe in the acute setting and there are limited biomarkers to identify which patients may develop drug-resistant epilepsy. As such, the development of T cell strategies would likely have a limited market and the focus should be on modulation of the myeloid compartment, including microglia, especially for patients with drug-resistant epilepsy.

Given the heterogeneous nature of epilepsy and the available preclinical models, the selection of appropriate animal models is crucial for vetting clinically relevant therapeutics (35). Immunological characterization, including the kinetics of the various preclinical epilepsy models, would provide necessary foundational knowledge for the field. A translational strategy to be considered includes complementary pharmacological and genetic knockout or overexpression of target(s) in the KA or pilocarpine models with initial screening of target-directed therapeutics in the acute electroshock models. Delivery of peripherally administered drugs to the CNS is hampered by the BBB, a common obstacle faced in developing neurologic therapeutics. However, disruption of the BBB has been shown in both patients with epilepsy and animal models of epilepsy (19, 150–154). Immunohistochemical analysis of tissue from patients with TLE has shown increased levels of CCL2 linked to BBB disruption and epileptogenesis (36, 152, 153). Therefore, systemic delivery of immunotherapies may be a viable strategy for these patients, presenting an advantage over treating other CNS diseases not accompanied by BBB disruption. If possible, the selection of targets in regions where BBB disruption is occurring may improve therapeutic responsiveness. The rapid emergence of pharmacological, ultrasonic, and mechanical means for opening the BBB could provide ways to acutely circumvent the BBB for strategies to treat epilepsy and other CNS diseases. Finally, the determination of the extent of BBB disruption and any alterations in the composition of vasculature in these epileptogenic regions is an area that needs future investigation.

## Conclusions

More recent profiling of human epilepsy specimens using more contemporaneous methodologies has identified an activated microglial population that may be interacting with T cells (20). Modeling epilepsy in preclinical murine models has suggested that CD8^+^ T cells may exert neurotoxic effects on hippocampal neurons, leading to TLE (38). While the immunosuppressive microenvironment in GRE should generally be beneficial if epilepsy has a proinflammatory immune landscape, patients with glioma have seizures. This apparent contradiction is partially resolved by the identification of glioma-elaborated or -induced factors, such as D-2HG and adenosine that are involved in seizure activity, but there are likely others. An improved understanding of the interplay between immune cells and neurons is required to help prioritize specific targets for the next generation of ASMs. Additionally, given the heterogeneity of epileptic etiologies, understanding differences between non-glioma epilepsy immune mechanisms and GRE mechanisms and other epilepsy syndromes is crucial to development of epilepsy subtype–specific therapeutics. For example, patients with IDH-mutant glioma may benefit from front-line IDH-mutant inhibitors to reduce seizure activity. Precision medicine strategies for epilepsy, rather than more generalized symptomatic control, may ultimately be of greater benefit to patients. Finally, cell type–specific therapeutics that target the myeloid compartment may emerge to prevent chronic epilepsy. Mouse models and human studies have provided important information regarding the contribution of the immune system to epileptic seizure developments; however, several key factors must be considered for future studies: (a) immune characterization and profiling of various types of epilepsy would be informative for the selection of immunomodulatory therapeutics, (b) preclinical murine models of epilepsy need to be aligned to clinical conditions to select and optimize the next generation of therapeutics, and (c) comprehensive metabolomic profiling should be conducted on epilepsy specimens to prioritize potential targeting strategies.

## Version 1 01/09/2024

Electronic publication
